# Incidents of Animal Life

**Published:** 1887-09-24

**Authors:** F. O. Morris

**Affiliations:** Rector of Nunburnholme, Yorkshire; author of 'A History of British Birds," etc.


### INCIDENTS OF ANIMAL LIFE.

COLLATED BY THE REV. F. O. MORRIS, B.A.,
Rector of Nunburnholme, Yorkshire; author of 'A History of British Birds," etc.

### ABOUT BIRDS.

I VENTURE to send you a little description of our birds. We have a small lobby between two doors—the outer front door, and a glass door leading into the hall. A pair of swallows have built there for two summers. Last year they made a nest just (inside the lobby) over the front door. We were constantly going in and out, and they took no notice of us. This year they returned, but instead of using their old nest, built one further in. We shut the front door till there were young birds, and the pair roosted in the lobby all night, flying out when the door was opened in the morning. Where they had hatched, we left the door ajar all night. They did not seem to be the least frightened or disturbed by our going into the lobby with a candle. When the young were flown they came back once or twice, and boldly flew right into the hall, and were twice found sitting on the top of my bed. I saw an account in *Chambers's Journal*, for September, of swallows building in a similar place, but the writer said they never came in unless he sat very still in a chair. Our swallows have flown in over our heads while we were standing in the doorway. Another curious thing happened this summer. One of the servants got up very early, about five o'clock, and on going into the passage saw a large brown owl sitting over my husband's dressing-room door. It must have flown in through an open window, and doubtless escaped in the same way. It was some hours in the house.

### MOTHER PARTRIDGE'S INSTINCT.

I venture to think that the accompanying account of the singular instinct of a partridge may be of interest. A friend of mine last summer was staying at Maidenhead. In the course of his visit a meadow was mown, and the men brought to view a partridge's nest, which they took to their master. The eggs being quite warm, it was decided to place them under a hen, who readily accepted the vicarious duty, and in due course the eggs were hatched. After the young birds were about six weeks old my friend and his host went to look at them, when the hen, apparently offended, went off, and would have no more to do with her nurslings. The young birds were left under the coop for that night, and next morning, when revisited, a hen partridge was with them.

### "A MUSICAL PARROT."

A singular illustration of the power of music upon what we (superior beings) are pleased to call "irrational creatures," was afforded by an anecdote I read in a Liverpool paper some time ago. A sailor belonging to that port had brought home a parrot from the Spanish Main, as a present to his wife and children; and Poll was usually hung outside the window to enjoy the sunshine, and see what was passing in the street. Some months after her arrival in her new home, a foreign-looking minstrel stopped one day before the house, and began singing some foreign air, to the accompaniment of a guitar. Poll listened attentively for some moments, keeping time with her head to the tune, and seeming to drink in every note. The music appeared evidently to recall to her memory

"The wild songs of her own native plains,
Every note that she loved awaking,"

and the recollection proved too keen for endurance, for after giving a few feeble flutters with her wings upon her perch, the poor bird fell dead from that perch heart-broken!

(*To be continued.*)

